# Circulating MicroRNAs: Potential and Emerging Biomarkers for Diagnosis of Human Infectious Diseases

**DOI:** 10.3389/fmicb.2016.01274

**Published:** 2016-08-15

**Authors:** Parmila Verma, Rajan K. Pandey, Priyanka Prajapati, Vijay K. Prajapati

**Affiliations:** ^1^Department of Biochemistry, School of Life Sciences, Central University of Rajasthan, AjmerIndia; ^2^Arya Kanya College, MeerutIndia

**Keywords:** biomarker, hepatitis, HIV, infectious diseases, miRNA, tuberculosis

## Abstract

MicroRNAs (miRNAs) are evolutionary conserved, small non-coding RNA with size ranging from 19 to 24 nucleotides. They endogenously regulate the gene expression at the post transcriptional level either through translation repression or mRNA degradation. MiRNAs have shown the potential to be used as a biomarker for the diagnosis, prognosis, and therapy of infectious diseases. Many miRNAs have shown significantly altered expression during infection. The altered expression of miRNA level in an infected human can be identified by the use of advanced diagnostic tools. In this review, we have highlighted the use of miRNA as an emerging tool for the identification of the human infectious disease. Till date, several miRNAs have been reported as a molecular biomarker in infectious diseases, such as miRNA-150 and miRNA-146b-5p in human immunodeficiency virus (HIV); miRNA-122, miRNA-21, and miRNA-34a in hepatitis; miRNA-361-5p and miRNA-29c in tuberculosis; miRNA-16 and miRNA-451 in malaria and miRNA-181 in *Helicobacter pylori* infection. The diagnosis of infection with the help of a biomarker is a non-invasive tool that has shown to have a key role in early diagnosis of infection. The discovery of circulating miRNA in the blood of infected patients has the potential to become a powerful non-invasive biomarker in coming future.

## Introduction

MicroRNAs (miRNAs) are evolutionary conserved, small non-coding RNA, playing a significant role in controlling human gene expression. They consist of 19–24 nucleotides long sequence and regulate nearly 30% of human gene expression at the post transcriptional level (Lewis et al., 2005; Farazi et al., 2008). Statistically, up regulation of single miRNA can regulate the activity of hundreds of genes (Lim et al., 2005; Grimson et al., 2007). Lin-4 was the first miRNA discovered in *Caenorhabditis elegans*, revealing its role in the transformation of L1–L2 larval stage and the normal adult structure development and its mutation leads to incapability for laying eggs (Chalfie et al., 1981; Feinbaum and Ambros, 1999). After 7 years of the discovery of Lin4, the second miRNA let-7 was discovered, again in *C. elegans*; which was associated with the developmental timing (Reinhart et al., 2000). MiRNAs play an extensive role in the maintenance of the regulatory signaling at the cellular level, which either provides protection from the disease or favors its persistency by inducing protected or unprotected signaling pathways, respectively (Wang et al., 2008).

**FIGURE 1:**
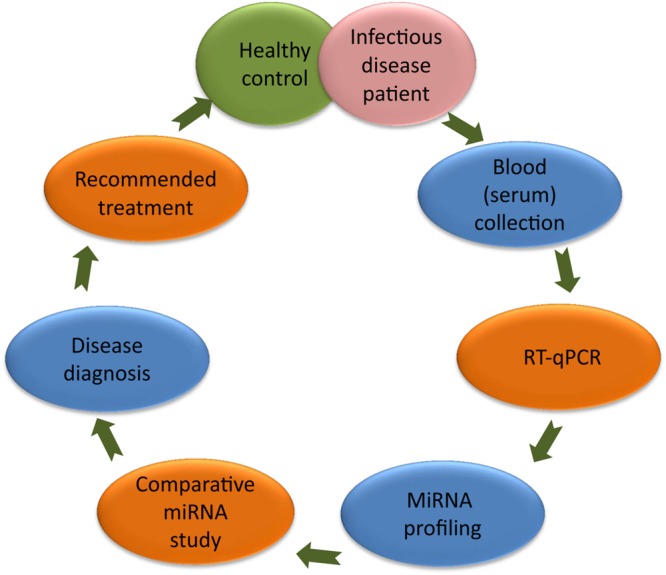
**MiRNA based diagnosis of human infectious diseases.** Colored ellipse represents the different steps of diagnosis. It include blood (serum) collection from healthy and diseased persons followed by RT-qPCR, miRNA profiling, comparative miRNA study leading to the diagnosis of disease. Later on, patient could be recommended for the available treatment.

Scientists have established a significant correlation between the miRNA and the cause of diseases (Pandey et al., 2016); therefore, today several miRNAs are being utilized as molecular biomarkers for the diagnosis of human infectious diseases (Almeida et al., 2011). During pathological conditions, alteration of a specific miRNA than healthy control can be used as a biomarker to predict the diseased condition (**Figure 1**). In this article, we have highlighted the importance of circulating miRNAs in the diagnosis of infectious diseases. In HIV infection, miRNA-223, miRNA-382, miRNA-125b, and miRNA-28, targets the 3′ untranslated region (UTR) of the HIV-1 messenger RNA, while miRNA-150 binds to Nef 3′ long terminal repeats (LTR) at 773 and 89 positions (Huang et al., 2007). In hepatitis, miRNA-122 expression target the 5′ end of hepatitis C virus (HCV) genome (Wahid et al., 2010). Altered expression of miRNA-365, miRNA-483-5p, miRNA-22, miRNA-29c, miRNA-101, and miRNA-320 are reported in tuberculosis and affect the mitogen-activated protein kinases (MAPK) and transforming growth factor beta (TGF-β) signaling to develop tuberculosis infection (Zhang et al., 2013). However, significantly lower plasma level of miRNA-16 and miRNA-451 has been reported in malaria patients (Chamnanchanunt et al., 2015). MiRNA-181 targets 3′UTR region of tumor suppressor Krüppel-Like Factor (KLF) encoding gene during *Helicobacter pylori* infection (Zabaleta, 2012). These altered miRNAs levels in human blood/serum during different pathogenic infections can be used as a biomarker in discrimination of human infectious diseases.

## Biogenesis of miRNA in Human Infectious Diseases

When hitherto infected vector bites to human, entry of a number of pathogens in the human body takes place. As the pathogen enters, they become active in the peripheral blood and releases several associated factors for their protection. In response to pathogenic activity several changes occur in the cellular system; simultaneously, leading to the production of the miRNAs controlling the diseased condition through cellular signaling. During the miRNA development process, the non-coding part of the host genome is transcribed by RNA polymerase II to form the primary miRNA having a local hairpin structure. Further, cropping of pri-miRNA by DROSHA (RNase III protein) leads to the formation of precursor (pre) miRNA. Pre-miRNA exported to the cytoplasm where it further processed by Dicer (RNase III type endonuclease) to form an RNA duplex which is subsequently loaded onto AGO1-4. Later on, passenger strand of RNA duplex is discarded to form mature miRNA in complex with AGO proteins (Ha and Kim, 2014). The resulting miRNA binds to the mRNA sequence and inhibits the translation process or degrades the target mRNA (Bartel, 2004; MacFarlane and Murphy, 2010).

## Research Studies on miRNA in Infectious Disease

This review highlighted the importance of miRNAs in the diagnosis of selected human infectious diseases caused by virus, bacteria and parasites (**Table 1**) and the respective way of diagnostics (**Figure 1**).

**Table 1:** Differential miRNA expression pattern in different infectious diseases.

S. No.	Infectious disease	Upregulated miRNA during infection	Downregulated miRNA during infection	Reference
1	HIV/AIDS	miRNA-382, miRNA-125b, miRNA-28, miRNA-21, miRNA-122, Let-7, miRNA-34a, miRNA-12, miRNA-19, miRNA-92b	miRNA-146b-5p, miRNA-223, miRNA-150, miRNA-16, miRNA-191, miRNA-17-5p, miRNA-20	Huang et al., 2007; Houzet et al., 2008; Dai, 2011; Munshi et al., 2014; Thapa et al., 2014
2	Hepatitis	miRNA-122, miRNA-34a, miRNA-101, Let-7b, miRNA-196, miRNA-448, miRNA-149, miRNA-638, miRNA-199a, miRNA-491	– – – – – – – – – –	Cermelli et al., 2011; Fu et al., 2013; Conrad and Niepmann, 2014
3	Tuberculosis	miRNA-144, miRNA-365, miRNA-146	miRNA-361-5p, miRNA-889, miRNA-576-3p	Qi et al., 2012; Yi et al., 2012; Song et al., 2015
4	*Helicobactor pylori* infection	miRNA-223	miRNA-129-1-3p, miRNA-129-2-3p	Li et al., 2012; Sandoval-Borquez et al., 2015
5	Malaria	miRNA-223, miRNA-19b	miRNA-451, miRNA-16	Goo et al., 2014; Chamnanchanunt et al., 2015
6	Trypanosomiasis	miRNA-193b, miRNA-338	miRNA-199a-3p, miRNA-27b, miRNA-126	Lueong et al., 2013; Lueong, 2014

### Human Immunodeficiency Virus (HIV) Infection

Human Immunodeficiency Virus infection leads to the development of an immunocompromised condition known as Acquired Immune Deficiency Syndrome (AIDS). HIV, targets to suppress cell mediated immunity, which leads to the decreases in number of CD4^+^ T lymphocyte cells and finally, deterioration of the immune power. HIV infected individuals have shown varying expression of host specific miRNAs along with viral miRNAs, which inhibit the post transcriptional gene regulation in host cell (Bartel, 2004; Soifer et al., 2007). Research, analysis, suggests that 62 different types of miRNAs were present in peripheral blood mononuclear cells (PBMCs) of HIV/AIDS infected human and among them only three miRNAs were up regulated whereas the remaining 59 miRNAs were found to be down regulated. Interestingly, miR-146b-5p, miR-223, miR-150, miR-16, and miR-191 among the down regulated miRNAs were found to be plentifully expressed in B and T-lymphocyte, confirming a positive disease status (Houzet et al., 2008). In HIV infection, increased expression of miRNA-223, miRNA-382, miRNA-125b, and miRNA-28 targets the 3′ UTR of HIV-1 mRNA, which may subsequently decrease HIV replication. MiRNA-150 binds to Nef 3′-LTR at 773 and 89 position to reduce the expression of PBMC oploid individuals. MiRNA-223 interacts with Nef 3′-LTR at 408^th^ residues of viral protein (Huang et al., 2007). The PBMC miRNA-146b-5p and miRNA-150 level was decreased in HIV/AIDS patient and an individual with antiretroviral therapy (ART) resistance while restoring with ART. The decreased levels of miRNA-146b-5p, miRNA-16, miRNA-191, miRNA-150, and miRNA-223 in PBMC compared to healthy individual provide the evidences for HIV infection (Houzet et al., 2008). However, the higher expression level of miRNA-21, miRNA-122 and lower expression level of miRNA-223 can be utilized to discriminate between HIV positive and HIV negative humans (Thapa et al., 2014). MiRNA-17-5p and miRNA-20 down regulate the expression of p300/CBP-associated factor (PCAF) histone acetyl-transferase, can induce the inhibition of the HIV virus in the human body (Munshi et al., 2014). MiRNA-222 can be used to distinguish diffuse large B cell lymphoma or primary central nervous system lymphoma (PCNSL), if the HIV-1 infected individual is not affected by AIDS-NHL (Non-Hodgkin’s Lymphoma; Thapa et al., 2014). HIV suspected individuals have shown the higher level of miRNA-21 compared to healthy control, which is also up regulated in activated B cells, and can be used to identify AIDS related NHL infection. The up regulation of let-7, miRNA-34a, and miRNA-12 provides a suitable environment to propagate the HIV infection and can also be used as a biomarker. MiRNA-29a and miRNA-29b targets the Nef 3′LTR/UTR viral gene at the 420 position and downregulates nef protein expression (Ahluwalia et al., 2008; Sun et al., 2011). HIV-infected PCNSL patient has shown a significantly increased level of miRNA-19, miRNA-21, and miRNA-92b in cerebro-spinal fluid (CSF; Dai, 2011) therefore, these miRNAs can also be used as a biomarker.

### Hepatitis

Hepatitis is a viral infection of human liver that results in swelling and inflammation. The symptoms include nausea, vomiting, weight loss, abdominal pain, jaundice, dark yellow urine, and fever. Acute hepatitis never show symptoms in the early stage and may change into chronic hepatitis leading to liver fibrosis, liver scarring, liver cancer, and increased level of hepatocellular carcinoma (HCC). The hepatitis virus can increase the level of a specific miRNA in the affected person’s blood/serum. At present, several miRNAs namely miRNA-122, miRNA-34A, miRNA-16, miRNA-21 have been reported as biomarkers in hepatitis related HCC (Cermelli et al., 2011). MiRNA-122 and miRNA-34a were significantly up-regulated while the miRNA-21 level was found to be normal during HCV infection.

Hepatic fibrosis, supported by the TGF-β signaling, promotes expression of miRNA-21 but the decreased expression of miRNA-21 suppresses the SMAD7 signaling. Therefore, miRNA-21 might be a useful biomarker in hepatic fibrosis infection (Bihrer et al., 2011). Secretions of enzymes and TGF-β from liver cells are associated with higher expression of miRNA-34a and miRNA-122, found to be helping in fibrosis development of liver tissue. MiRNA-122 specifically binds to 5′ end of HCV genome into the liver and encourages viral replication and progression of infection. The expression of miRNA-101 was high in HCC infected individuals, can give the information of hepatitis-B surface antigen in the liver (Fu et al., 2013). MiRNA-122, miRNA-199a, miRNA-196, miRNA-448, and let-7b was found to be expressed during hepatitis C infection and regulate the pathogenicity. Circulating Let-7b, interacts with the conserved NS5B coding sequence and 5′ UTR region of the HCV genome, leading to the inhibition of replication (Cheng et al., 2012). MiRNA-196 and miRNA-448 were found to be up-regulated in HCV infected individuals, target the coding region (CORE and NS5A) of HCV genomic RNA. Simultaneously, miRNA-149, miRNA-638, and miRNA-491 were up-regulated due to HCV infection and enhance the viral replication by inhibiting the AKT/PI3 kinase (Conrad and Niepmann, 2014). Therefore, these miRNAs show the great potential for their utilization as molecular markers to diagnose of hepatitis viral infectious disease by non-invasive methods.

### Tuberculosis

Tuberculosis is a contagious and infectious disease caused by *Mycobacterium tuberculosis* bacteria; which affects the human lungs. It is of two types; latent or active tuberculosis. If a person is infected with *M. tuberculosis* but lacks the symptoms and do not feel sick, called latent tuberculosis, but a person with *M. tuberculosis* infection and its respective symptoms, represents active tuberculosis. Recent research on tuberculosis significantly offers beneficial information about miRNAs as a biomarker for the investigative purposes. Pulmonary tuberculosis infected individuals have shown elevation in 92 miRNAs in serum. Among which 59 miRNAs were up regulated and rest of 23 miRNAs were down regulated as compared to healthy controls (Yi et al., 2011). MiRNA-144 was found to be highly expressed in the PBMC compared to miRNA-361-5P, miRNA-889, and miRNA-576-3p that were significantly down regulated in active tuberculosis patients. However, increased level of miRNA-361-5p, miRNA-889, and miRNA-576-3p have been reported in tuberculosis infected serum as non-invasive molecular biomarker for rapid diagnosis and prevention of tuberculosis infection (Qi et al., 2012). Alteration in miRNA-378, miRNA-483-5p, miRNA-22, miRNA-29c, miRNA-101, and miRNA-320 are specific for pulmonary tuberculosis and non-tuberculosis infections. These miRNAs are deferentially expressed and affect MAPK & TGF-β signaling, which can be used for the identification of tuberculosis infection. MiRNA-378 and miRNA-101 target the MAPA1 signaling while miRNA-483-5p, miRNA-320, miRNA-22 affect AKT-3, and BCL9L signaling to develop tuberculosis infection (Zhang et al., 2013). Upregulated miRNA-365 has shown inhibitory effect against IL-6 signaling by binding at its 3′UTR in tuberculosis infection (Song et al., 2015). MiRNA-146 has shown 3.34 fold over expression as compared to healthy individuals (Yi et al., 2012). Researchers have identified specific miRNAs (miRNA-29a and miRNA-22), that were used to discriminate between active and latent tuberculosis infection (Zhang et al., 2013). MiRNA-29 was considered to control the innate and acquired immune response by targeting IFN-γ in pulmonary tuberculosis (Wiwanitkit and Wiwanitkit, 2012) therefore, nominated as a biomarker. These miRNAs can be used as a molecular biomarker for the diagnostic and prognostic by the Taqman Low Density Assay (Qi et al., 2012).

### *Helicobacter pylori* Infection

*Helicobacter pylori* are spiral shaped bacteria that mainly grow inside the gastrointestinal tract and is capable of infecting the inner lining of the stomach. Generally, infection of *H. pylori* is harmless, but in some cases it is responsible for the ulcer of stomach and small intestine. *H. pylori* infection causes ulcer, nausea, vomiting, unexpected weight loss and abdominal pain. However, some other facet of *H. pylori* infection was also reported that its colonization of the stomach is the major cause of gastric cancer and gastric mucosa associated lymphoid tissue (MALT; Parsonnet et al., 1991; Eslick et al., 1999; Uemura et al., 2001). MiRNAs have also shown their role in gastric cancer and have been reported as a potential molecular biomarker (Zabaleta, 2012). MiRNA-223, miRNA-22, miRNA-218, and miRNA-25 has been found to be associated with metastasis and gastric cancer. MiRNA-223 over expression represses the gastric cancer and inhibits the exosomes transfer of metastatic cells in another part of the body. MiRNA-218 has found to be lowering the cell development and invasion as compared to healthy control (Li et al., 2012). MiRNA-181 interacts with the 3′ UTR region of the tumor suppressor KLF gene and have ability to inhibit the apoptosis of tumor cells (Zabaleta, 2012). Current research analysis on miRNAs (miRNA-10b, miRNA-21, miRNA-223, miRNA-338, let-7a, miRNA-30a-5p and miRNA-126) in gastric cancer have been noted to show a positive correlation with the identification and diagnosis of early cancer stages (Li et al., 2012). Knockdown of the miRNA-21 promotes the apoptosis and reduced cell proliferation (Zhang et al., 2008). MiRNA-129-1-3p and miRNA-129-2-3p have shown down-regulation in gastric cancer patient as compared to the healthy control therefore these miRNAs were responsible for the cell proliferation and cell cycle. Furthermore, altered expression of 16 miRNAs was reported on *H. pylori* mediated gastric cancer condition. Altered expression of miRNA-221, miRNA-744, and miRNA-376c significantly give a positive identification of the disease (Sandoval-Borquez et al., 2015). MiRNA-142-5p and miRNA-155 have shown their role as a molecular biomarker for gastric MALT (Saito et al., 2012). On the basis of above reports, we can conclude that these aforementioned miRNA can be used as a potential biomarker for the identification of the *H. pylori* mediated gastric cancer and gastric MALT.

### Malaria

Malaria is a vector borne infectious disease caused by protozoan parasites namely *Plasmodium vivax, P. faliciparum, P. malariae, P. knowlesi, and P. ovale*. Among them *P. falciparum* is considered to be more fatal as it leads to cerebral malaria, if proper medication has not been given. Malarial parasite causes high fever, headache, extreme tiredness, clogging blood vessels, vomiting, and rupture of blood vessels. Since, the symptoms appear after 15 days of infection; therefore we cannot diagnose early infection by available molecular tools. Infection of the malarial parasite in human could potentially alter the expression of erythrocytic miRNA in the blood and these miRNAs can be diagnosed by the molecular diagnostic tool like quantitative reverse transcriptase polymerase chain reaction (RT-qPCR), microarray profiling and next generation sequencing can give an indication of infection. MiRNA-451 and miRNA-16 have been found to be down regulated in blood/serum of malaria patients as compared to healthy control. The first reason behind the downregulation of miRNA is the degradation of red blood cells (RBC) miRNA after the *Plasmodium* infection (LaMonte et al., 2012). The second reason behind this downregulation is the clearance of miRNA in case of hypersplenism during malaria infection, therefore it may increase the RBC destruction by the spleen (Buffet et al., 2011). Furthermore, lower expression of these two miRNAs (miRNA-451 and miRNA-16) significantly gives information about the parasite load in the blood (Chamnanchanunt et al., 2015). Sickle cell erythrocyte infected person may have higher expression of the miRNA-451 and let-7i in blood (Eridani, 2013). Healthy person infected by malaria parasite has shown higher expression of miRNA-223 and miRNA-19b in normal RBC cells in blood (Goo et al., 2014).

### Trypanosomiasis

Trypanosomiasis (sleeping sickness) is an infectious disease caused by protozoan parasitic of genus *Trypanosomes*. The control of trypanosomiasis infection cannot be done by early diagnosis as symptoms do not appear generally in the early stages of infection. Levels of miRNAs (miRNA-199a-3p, miRNA-27b, and miRNA-126) have been noted significantly decreased in humans infected by *Trypanosoma brucei gambiense* as compared to healthy individuals. These miRNA deregulate the signaling of toll like receptor and NF-kB after the infection of *Trypanosoma* (Lueong, 2014). Therefore, the molecular biomarker such as miRNAs can be used to identify the infection of the trypanosomes or sleeping sickness using peripheral blood leukocytes. However, trypanosomiasis patients have shown the higher expression level of miRNA-193b and miRNA-338 compared to control (Lueong et al., 2013). At present time available molecular tools are very invasive, less sensitive for detection of trypanosomiasis infection. So by using miRNA based molecular biomarker, one can give significant positive information about the infection at the early stage.

## Challenges of miRNA as a Molecular Biomarker

Presently, only few standardized procedures are available for the isolation and characterization of specific miRNA. Experimental research and its observation have shown that small interfering RNA, premature miRNAs, and transfer RNA may interfere with specific miRNA during the process of isolation and characterization. Therefore, this interference leads to the false positive result, which should be taken care during diagnosis. Small non-coding miRNA needs a carrier molecule for extraction, such as long RNA for precipitation of particular miRNA. The necessity of the large amount of RNA input for the northern blot technique can generate difficulties in the quantification of the miRNA. RT-qPCR, microarray profiling and next generation sequencing have been found to be useful for the identification of novel miRNA. Characterization of miRNA should be done by an experienced researcher having a good knowledge of molecular biology as well as Bioinformatics. The available tools of miRNA characterization are very expensive; therefore everyone cannot afford this diagnostic tool. During the course of the experiment one should need to take extra precautions to avoid experimental contamination and data interpretation issues which can alter the level of miRNA and can provide false results.

## Author Contributions

Conceived and designed the experiments- PV, RP, PP, and VP; Wrote the paper- PV, RP, PP, and VP.

## Conflict of Interest Statement

The authors declare that the research was conducted in the absence of any commercial or financial relationships that could be construed as a potential conflict of interest.
